# Public perspectives on tick bite exposure, healthcare visits and associated allergies in iberia

**DOI:** 10.1080/07853890.2025.2499028

**Published:** 2025-05-03

**Authors:** Rita Vaz-Rodrigues, Marta Rafael, Davide Carniato, José de la Fuente

**Affiliations:** a SaBio. Instituto de Investigación en Recursos Cinegéticos IREC-CSIC-UCLM-JCCM, Ciudad Real, Spain; b Department of Veterinary Medicine, University of Sassari, Sassari, Italy; c Department of Veterinary Pathobiology, Center for Veterinary Health Sciences, Oklahoma State University, Stillwater, OK, USA

**Keywords:** α-gal syndrome, allergy, citizen science, Iberian Peninsula, tick-borne diseases

## Abstract

**Background:**

Tick bites and tick-borne diseases (TBDs) are a worldwide concern, with growing evidence of an ongoing spread and emergence of new cases. This study applied a participatory citizen science across Spain and Portugal to gather public perspectives on the impact of tick bites, the presence of TBDs and a potential connection with the α-Gal syndrome (AGS).

**Methods:**

Data collected from the questionnaire (380 participants) was geographically represented using QGIS Geographic Information System and fitted into multiple generalized linear models (GLMs). Statistical analysis identified factors influencing the occurrence of local skin reactions post-tick bite, AGS-compatible symptoms and visits to health centers following tick exposure.

**Results:**

Results showed that the probability of developing localized skin reactions to tick bites rises with increasing age (*χ*^2^ = 0.006, *p* < 0.05), the occurrence of multiple bites (*χ*^2^ = 0.006, *p* = 0.063) and among individuals bitten in the center-north part of the peninsula (*χ*^2^ = 0.006, *p* = 0.058). Seeking medical care following tick bite was reported by 21.6% of respondents, being more common in first-time bite cases (*χ*^2^ = 0.002, p < 0.01), men (*χ*^2^ = 0.002, *p* < 0.01) or individuals presenting localized skin symptoms (*χ*^2^ = 0.002, *p* < 0.05). Although 38 inquiries (10.0%) showed signs of AGS, only 3 had received a formal diagnosis, with the odds of developing this disease marginally rising with advancing age (*χ*^2^ = 0.215, *p* = 0.066).

**Conclusions:**

This study provides valuable insights that should be considered to improve TBDs surveillance and diagnostic strategies, as well as developing preventive measures to reduce tick bite exposure and TBD cases.

**Key Messages**

- Developing local skin reactions to tick bites increases with age, multiple bites and in individuals bitten in central-northern Iberia.
- Seeking medical care following a tick bite is more common in first-time bite cases, men or individuals with local skin symptoms.
- Although 38 inquiries (10.0%) showed signs of AGS, only 3 were formally diagnosed, with risk increasing with age.

## Introduction

Ticks are one of the most ubiquitous ectoparasites found worldwide [1]. Unlike other hematophagous parasites, ticks have a unique life cycle that involves multiple hosts, including mammals, birds, reptiles and humans [2]. While most ticks represent no major harm to their hosts, bites from hard ticks (Ixodidae) may imply significant health issues with various symptoms ranging from localized skin reactions to systemic complications [3,4]. In addition, ticks act as vectors for pathogens, contributing to the spread of diseases with public health significance [4].

Tick bites in humans typically lead to an immediate local skin inflammatory reaction [5]. This process is orchestrated by an array of cytokines and immune cells, particularly monocytes, neutrophils and dendritic cells, which translates into redness, intense pruritus and occasionally localized oedema [3,6]. However, late symptoms at tick bite site may also appear and are generally linked to infectious agents potentially affecting human health [3]. Tick-bitten individuals may also develop systemic reactions including fever, headaches, fatigue, or muscle pain [7]. These generalized symptoms are particularly associated with tick-borne diseases (TBDs) such as Rocky Mountain spotted fever (*Rickettsia rickettsii*), or anaplasmosis (*Anaplasma phagocytophilum*) [3,6].

Furthermore, humans occasionally develop tick-borne allergies like the α-Gal syndrome (AGS), potentially prompted by the inoculation of several biomolecules from hard tick saliva, such as glycan α-Gal, protein allergen-like p23, metalloproteases, and prostaglandin E2, into the host’s system [8–10]. Although the complete immunological mechanisms are not yet fully understood, it is known that α-Gal sensitization leads to a Th2-skewed profile, mirrored by higher titers of specific anti-α-Gal IgE and cytokines like interleukins IL-4 and IL-13 produced by CD4+ T cells [11,12]. The AGS symptomatology is variable and appears after consumption of α-Gal positive mammalian-derived products [13]. Individuals may experience symptoms ranging from a delayed anaphylactic reaction 3 to 6 h post-meat consumption, to gastrointestinal complications including abdominal pain, reflux, emesis, and diarrhea, cardiorespiratory issues like dyspnea and hypoxia, and cutaneous manifestations such as pruritus, urticaria and angioedema [14,15]. Therefore, the wide range of symptoms and case-to-case variability, along with the limited awareness among healthcare professionals and general population affects diagnosis and treatment of AGS. Additionally, limited diagnostic capacities and lack of standardization in diagnostics often results in frequent underdiagnosis [16].

To address the challenges posed by tick bites in Spain and Portugal, herein we used citizen science to explore the development of local skin reactions following tick bites and potential subsequent onset of systemic symptomology after mammalian meat consumption, consistent with AGS. Moreover, our study aims to analyze spatial distribution of recorded tick bites in mainland Portugal and Spain, and further associations between triggered reactions with factors such as age, gender, number of bites and geographical region of bites. Predictors including age, gender, skin response and number of bites influencing visits to healthcare facilities after tick exposure were also analyzed.

## Methods

### Questionnaire and data collection

An anonymous single-response web-based questionnaire consisting of 16 open-ended, multiple choice and closed questions (https://forms.gle/wSZUeEiMt1ST3JvV9; Supplementary Data 1), collected data from September 2023 to February 2024 (6 months). The survey was run by Google Forms, whose purpose was to gather information regarding the effect of tick bites in the Iberian Peninsula (Spain and Portugal) and the possible connection to AGS. The questionnaire presented demographic questions such as age, gender and country of residence. Furthermore, data concerning geographical tick bite location (country and region) were also collected, along with information on the development of local tick bite reactions like redness, pruritus, inflammation and hives, or systemic reactions after the ingestion of mammalian meat. Systemic AGS-related symptomatology included cutaneous (pruritus, oedema and urticaria), cardiorespiratory (anaphylaxis, dyspnea and cardiovascular distress) and gastrointestinal (diarrhea, abdominal pain, vomit, and reflux) problems. The questionnaire also contained two questions to determine if the respondents visited a health center after tick bite and if a proper diagnosis was obtained later. An open-end question was included to allow respondents to provide additional information if needed. A question regarding participants consent was also present, ensuring all inquiries included in this study agreed with the use and publication of this anonymous information. Participants were adults (at least 18 years old) who voluntarily participated upon giving informed consent. On average, respondents took about 5 min to complete the questionnaire. The study adheres to the Declaration of Helsinki. Written consent was obtained from all participants included in the study and the use of individual data was approved by the Ethical and Scientific Committee (Health Service of Castile-La Mancha, SESCAM C-73, “Study of prevalence, risk factors and epidemiological, clinical, allergic and molecular characteristics of individuals sensitized to alpha-Gal”).

During the 6 months period, a total of 593 answers were collected, with 410 respondents (69%) reporting having been bitten by a tick. Among these, 30 were bitten outside Spain’s or Portugal’s mainland, leaving 380 cases for analysis (23 were bitten in Portugal, 352 in Spain and 5 in both countries; Supplementary Data 2). The demographic information of the final sample is disclosed in Supplementary Tables 1 and 2.

**Table 1.** Details on reported cases of diagnosed α-gal syndrome (AGS).

Gender	Age	Number of tick bites	Location of tick bite	Local skin reactions to tick bite	Systemic reactions to mammalian meat intake
Female	18	Single	Basque Community (Spain)	Multiple (redness and pruritus)	Multiple (respiratory distress, pruritus and abdominal pain)
Male	50	Single	Principality of Asturias (Spain)	None	Multiple (pruritus, diarrhoea and reflux)
Male	60	Single	Principality of Asturias (Spain)	Multiple (redness and pruritus)	Multiple (pruritus and anaphylactic allergic reaction)

### Spatial and statistical analysis

Spatial distribution of the respondents was obtained using the Nomenclature of Territorial Units for Statistics (NUTS) code number 2 for mainland Spain (autonomous communities) and mainland Portugal (statistical regions), referring to the administrative divisions of these countries. From the final sample used to show demographic information, 57 Spanish cases were removed due to lack of response, leaving a total of 323 cases for mapping. Relevant variables such as number of tick bites, tick bite local cutaneous reactions and systemic symptoms after mammalian meat consumption were registered at NUTS2 level. Data on local skin reactions to tick bite were overlaid on percentage of health center visits after tick exposure. Data on development of systemic symptomatology after the consumption of mammalian meat after tick bite was spatially overlapped with the percentage of multiple tick bites registered. The results obtained were depicted using the open-source software QGIS Geographic Information System (version 3.34 *Prizren*, QGIS Association).

To analyze the most determinant factors influencing the presence of local skin reactions after tick bite and the development of systemic symptomatology after the consumption of mammalian meat post-tick bite, we fit a generalized linear model (GLM) with a binomial regression and logit link function. Local skin reactions and systemic symptoms were set as binomial response variables in two different models using the *glm* function from the *lme4* package [17]. Predictors (fixed factors) included in the models were age, gender, number of tick bites, and the geographical location of the bites (center-north or center-south regions, Supplementary Table 3). The relationship between the outcome variable (presence/absence of local or systemic reactions) and the predictors can be expressed as: outcome variable ∼ age + gender + tick bites + location. Therefore, the study was not focused on etiology but rather on investigating which predictors may be associated with the development of the most common on-site post-tick bite signs such as redness, pruritus, inflammation and hives and without including systemic symptomatology following tick bite for analysis. The development of AGS-related systemic symptomatology is IgE-mediated and does not occur immediately after a tick bite, which only causes sensitization, but usually between 2 and 6 h after the consumption of mammalian meat or derived bioproducts. Accordingly, in the questionnaire respondents were asked about previous history of tick bites (question No. 4) and development of allergies in the bitten site (question No. 11) and/or after tick bite and 1–6 h after consumption of red meat (question N. 12) (Supplementary Data 1). The local reaction model comprised 278 respondents, whereas the systemic reaction model included 286 individuals. Another GLM with a logit link was fitted to study the effects of age, gender, localized skin reactions and number of tick bites on visits to healthcare centers after tick exposure. This model comprised 314 individuals for analysis. Models fit were evaluated based on predictive capacity, normality presence and lack of residual patterns in data variation. The plots obtained were created using the *ggplot2* package [18]. All the analysis were performed using the R software, version 4.4.0 [19]. Statistical significance was declared using a *p*-value lower than 0.05 with a confidence level (CL) of 95%.

## Results

### Descriptive trends of tick bites

**Figure 1.**
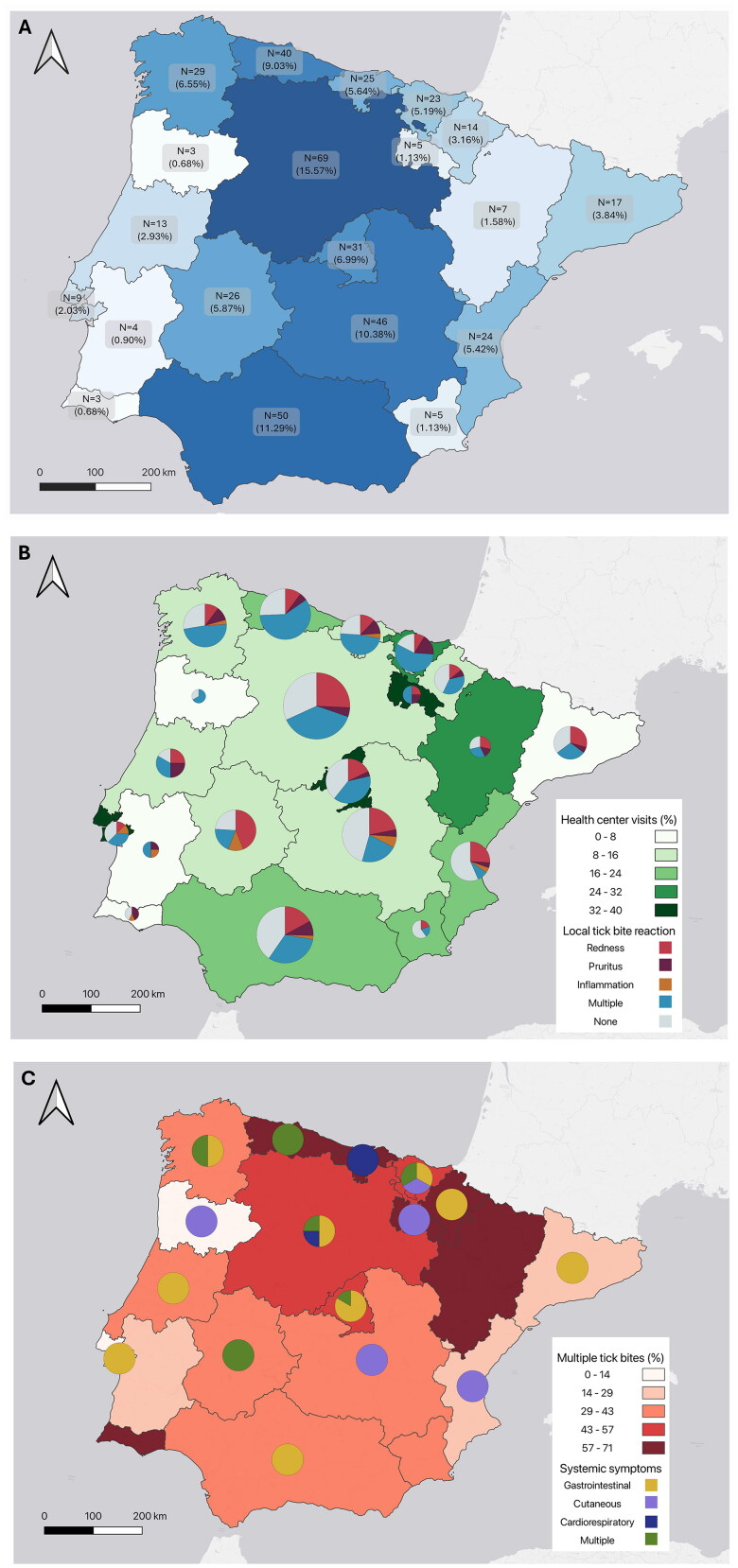
Geographically mapped questionnaire data on tick-bite exposure and effects across mainland Spain and Portugal. (a) Number and percentage of total tick bites recorded per autonomous community in mainland Spain and statistical region in mainland Portugal and color coded with a blue gradient to enhance visualization. Data includes a total of 443 tick bites over 323 individuals. (b) Cutaneous local tick bite reactions over the percentage of visits to health centers after tick exposure. Focal immediate tick bite reactions at skin level are represented with a pie chart and include single reactions, plus the combination of two or more single reactions (multiple) and the absence of reactions (none). The size of each chart is proportional to the number of inquiries. (c) Systemic α-Gal syndrome (AGS)-compatible symptoms overlapped with the percentage of multiple tick bites. AGS-like symptoms are depicted with a pie chart and include single reactions like gastrointestinal signs (diarrhea, abdominal pain, vomit and reflux), cutaneous reactions (pruritus) and cardiorespiratory problems (allergy and cardiovascular distress). The occurrence of two or more of these symptoms is compiled in the multiple section. The absence of systemic reactions was not added to the map and is reported in Supplementary Figures 1 and 2. Administrative divisions with no pie chart showed absence of systemic reactions post-meat consumption.

Overall, men accounted for the majority (*N* = 208/380, 54.7%) of the sampled respondents bitten by ticks (Supplementary Table 1). The age distribution of the sampled inquiries is detailed in Supplementary Table 2, being the 45 to 54 age group the most represented (*N* = 103, 27.1%), followed closely by the 25 to 34 age group (*N* = 98, 25.8%). Despite being exclusively tick-bitten in mainland Spain and Portugal, the participants presented different places of residence. Territorial distribution accounted for 352 individuals (92.6%) living in Spain, 22 (5.9%) residing in Portugal and 6 (1.5%) in other countries. Over the 323 regionally mapped responders, a total of 443 tick bites were reported. Distribution of registered tick bites per autonomous community in mainland Spain and by statistical regions in mainland Portugal is shown in Figure 1a.

### Local skin reactions to tick bites are affected by age, number and region of bites

**Figure 2.**
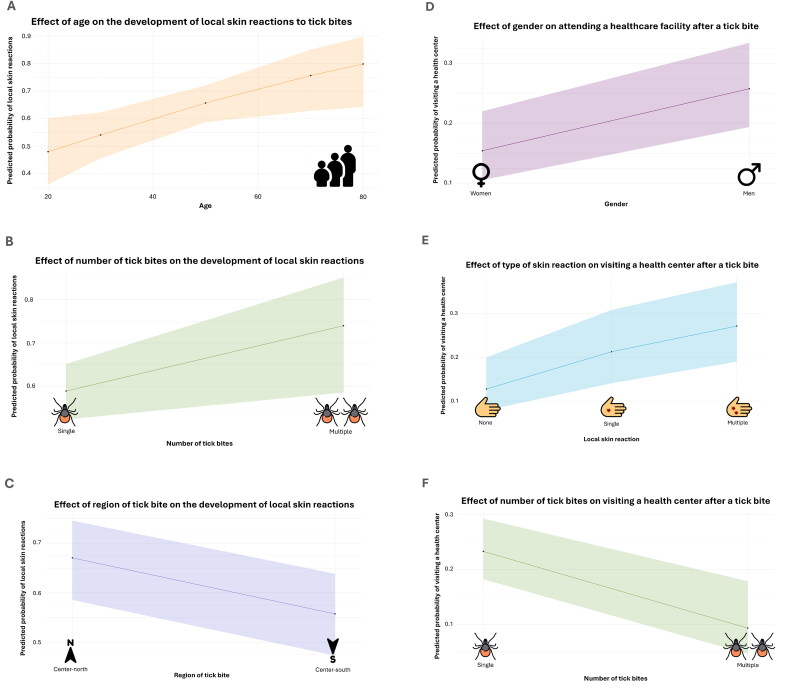
Predictive factors influencing local skin reactions and health center visits following tick exposure. Effect of age (a), number (b) and region (c) of tick bite on the development of local skin reactions and the effect of gender (d), type of local skin reaction (e) and number of tick bites (f) on attending a healthcare facility after tick exposure. The predicted probability of each outcome variable was determined by fitting a generalized linear model (GLM) with a binomial regression and logit link function.

Around 51.3% (*N* = 195/380) of the individuals bitten by ticks developed immediate focal skin redness, while 31.6% (*N* = 120/380) experienced pruritus, and 26.3% (*N* = 100/380) showed signs of cutaneous inflammation. Single reactions were observed in 30.3% (*N* = 115/380) of sampled individuals, whereas 32.4% (*N* = 123/380) displayed multiple reactions and 35.0% (*N* = 133/380) of the cases presented no skin reaction. GLM results indicated that age is a significant factor in the development of cutaneous reactions to tick bites (*χ*^2^ = 0.006, *p* < 0.05), with the odds of these symptoms increasing by 2% for each additional year of age (Figure 2a). Furthermore, individuals who were bitten multiple times showed a marginally significant increase in the likelihood of developing immediate skin reactions (*χ*^2^ = 0.006, *p* = 0.063, Figure 2b), a tendency also observed in those bitten in the center-north part of the peninsula (*χ*^2^ = 0.006, *p* = 0.058, Figure 2c). No significant differences were found between genders. The numerical output of the statistical model is in Supplementary Table 4.

### Seeking medical attention after a tick bite depends on gender, skin response and frequency of bites

Mapping of local symptoms per administrative division was combined with the percentage of visits to health centers after tick bite (Figure 1b). Interestingly, Iberian metropolitan areas of Lisbon and Madrid displayed the highest percentages of healthcare facility visits with 33.3% and 32.2%, respectively. Overall, only 21.6% (*N* = 82/380) of individuals received care at a health center after a bite. Interestingly, GLM results showed that men were more likely to seek medical care than women (*χ*^2^ = 0.002, *p* < 0.01, Figure 2d). Moreover, the predicted probability of attending a healthcare facility rises significantly when individuals exhibit any single form of skin reaction, becoming this increase even more pronounced in cases of multiple reactions (*χ*^2^ = 0.002, *p* < 0.05, Figure 2e). Respondents bitten for the first time by ticks are 2.95 times more likely to consult a healthcare professional compared to those who have already experienced multiple bites (*χ*^2^ = 0.002, *p* < 0.01, Figure 2f). Age did not affect the probability of seeking medical care after a tick bite. The numerical output of the statistical model can be found in Supplementary Table 5.

**Figure 3.**
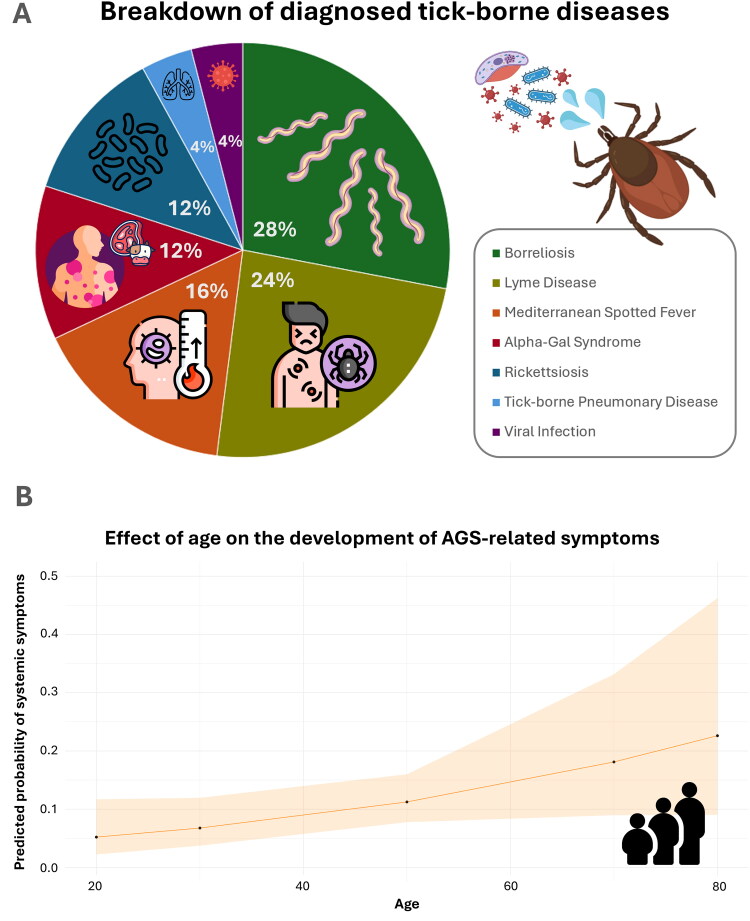
Tick-borne diseases (TBDs) diagnosis and age effect on the α-gal syndrome (AGS)-compatible symptoms. (a) Breakdown of TBDs diagnoses from inquiries who attended a health center post-tick bite. (b) Effect of age on the development of systemic symptoms, compatible with AGS, after mammalian meat intake and post-tick bite. The predicted probability of presenting systemic symptoms (cutaneous, gastrointestinal and cardiorespiratory) was determined by fitting a generalized linear model (GLM) with a binomial regression and logit link function.

Roughly 30.5% (*n* = 25/82) of inquiries who attended a health center were diagnosed with a TBD, as detailed in Figure 3a. Patients without a TBD diagnosis (69.5%, *n* = 50/82) underwent tick removal and, in some cases, blood tests were conducted. Occasionally, systemic antihistamines were prescribed.

### The development of AGS-compatible symptoms is influenced by age

Results on systemic symptomatology after mammalian meat intake and post-tick bite revealed a total of 38 cases (10.0%) displaying one or more AGS-compatible symptoms. These included cutaneous (pruritus, oedema and urticaria), cardiorespiratory (anaphylaxis, dyspnea and cardiovascular distress) and gastrointestinal (diarrhea, abdominal pain, vomit and reflux) problems. Specifically, 42.1% (*n* = 16/38) showed signs of cutaneous manifestations, 21.1% (*n* = 8/38) of cardiorespiratory distress and 65.8% (*n* = 25/38) presented gastrointestinal problems. A single symptom was present in 71.1% (*n* = 27/38) of the cases, while two or more symptoms were observed in 28.9% (*n* = 11/38) of the stratified inquiries. Territorial distribution of these symptomatology, overlapped with the percentage of multiple tick bites can be observed in Figure 1c. Furthermore, the odds of developing systemic reactions to meat consumption after a tick bite tend to marginally increase by roughly 3% for each additional year of age (χ^2^ = 0.215, *p* = 0.066, Figure 3b). Other predictors like gender, number and region of tick bites were not significant. The numerical output of the statistical model can be found in Supplementary Table 6. On the other hand, 88.2% (*N* = 335/380) of our respondents did not show any AGS-related symptoms (Supplementary Figures 1 and 2). Notably, of the 28 cases compatible with AGS, only 3 individuals were diagnosed with the disease. Detailed information on each case is in Table 1.

## Discussion

Tick bites and TBDs are a worldwide concern, with growing evidence of an ongoing spread and emergence of new cases driven by social-behavioral factors and current climate changes [20,21]. This study focused on describing local cutaneous reactions to tick bites and AGS-related symptomatology, while exploring their associations with factors such as age, gender, number and region of bites in mainland Spain and Portugal. Additionally, this research investigated demographic and epidemiological trends in health center visits after a tick bite, along with a detailed overview of the detected TBDs.

Ticks, as hematophagous pathogens, embed their mouthparts and inject saliva into human skin, sometimes triggering acute localized reactions [3,22]. We reported redness as the most predominant dermatological finding developed after a tick bite, followed by pruritus. A similar reaction pattern was observed in tick-bitten individuals from an investigation conducted in south-eastern Poland [23]. In fact, skin redness is one of the most common responses to several different external stimuli, which can be accompanied or not by allergen hypersensitivity [24]. When skin is repeatedly exposed to the same noxious stimuli such as a tick bite, cutaneous response may intensify due to sensitization that leads to an exacerbated immune response, with further activation of the inflammatory cascade [25]. This aligns with our findings regarding the connection between multiple tick bites and a higher probability of developing localized immediate skin reactions. Aging is also an underlying factor associated with immunomodulatory changes that increase the risk of higher intensity and duration of tissue damage, allergic and inflammatory processes [26,27]. Our results are corroborated by these findings and indicate that the odds of exhibiting cutaneous reactions to tick bites increase by 2% for each additional year of age. Skin symptoms at tick bite site were heightened in the center-north region of the study area, possibly due to geographical differences in tick species distribution. The primary vector of tick-borne zoonosis, *Ixodes ricinus*, presents a more environmental favorability for the northern third of the Iberian Peninsula and shows moderate to low abundances in southern peninsular areas [28,29].

Tick bite incidence is challenge to quantify, as mild symptomatic cases often remain unreported, and only a small fraction of individuals seek medical attention following a tick encounter [30]. Our findings report that 21.6% of respondents sought care at a health center after a bite, with slightly higher numbers encountered in the metropolitan areas of Lisbon (33.3%) and Madrid (32.2%). In fact, urbanization levels have been linked with health center visits through sociodemographic characteristics of the residents, determined by higher service availability and financial ability to access healthcare [31]. Men were more likely than women to seek medical care, a pattern equally observed in a 2017–2019 study in the United States on emergency department visits for tick bites [32]. Notably, several studies indicate that the general gender trend for utilizing health care services is inversed, with women presenting a higher number of primary care visits than men [33,34]. After all, seeking medical attention is a complex process that involves structural and individual factors like socioeconomic disparities, cultural norms and level of education [34]. First-time tick bite cases were nearly 3 times more likely to consult a healthcare professional than those with a history of previous bites. Presumably, first-time cases lack knowledge regarding tick removal techniques and symptom monitoring protocols. Individuals who have experienced multiple tick bites are often more informed and confident to manage the situation, reducing the perceived need for medical intervention. This disparity in behavior highlights the importance of educational interventions about when to seek medical care for tick bites, especially for inexperienced individuals [35].

In determining the best-fitting models, several predictors which do not enter the final model, are considered and selected through stepwise algorithms. This approach allows us to identify the most important predictive variables, those that explain the greatest variance in our response variable. In this sense, the model with the lowest AICc for cutaneous reactions was “Outcome Variable ∼ Age + Gender + Tick Bites + Location” and for healthcare visits was “Outcome Variable ∼ Age + Gender + Localized Skin Reactions + Number of Tick Bites.” Considering the predictors associated with each model, it can be possible that age influences the development of skin reactions, but localized skin reactions being a stronger predictor of whether medical care is sought. The healthcare visit model is probably capturing this more direct link between the reaction and seeking care, whereas the cutaneous reaction model focuses on factors (like age) that influence the occurrence of the reaction itself. The key takeaway is that age may influence the occurrence of reactions, but localized skin reactions are the critical factor in seeking medical care. Furthermore, there could also be interactions between age and localized skin reactions that are not captured in the models. For example, older individuals may experience milder skin reactions and therefore are less likely to seek medical attention, even though they may still develop the reaction. Another potential explanation is that younger individuals may be more likely to seek medical care due to greater concern about symptoms or perceived severity.

Immunomodulatory effects caused by tick salivary biomolecules can lead to the development of tick-borne allergies like AGS [25,36]. The overall prevalence of sensitization to α-Gal in Spain was 8.1% in 2014 [37] and 15.7% by 2019, with northern Spain showing higher prevalence rates [38]. We identified a total of 38 respondents (10.0%) exhibiting one or more AGS-compatible symptoms, but only three individuals with a formal diagnosis, all of whom were bitten in the Northern area of Spain. Such values disparities could mean that not only AGS may be underdiagnosed [16], but also that diagnostic challenges create a gap between symptom presentation and formal diagnosis. A contributing factor to this issue is the highly variable and individual-specific nature of AGS symptomatology [10]. Our findings reveal that 44.8% of potential AGS cases manifested exclusively gastrointestinal signs, a presentation that can overlap with other conditions, especially if the patient’s anamnesis is poorly documented. A recent study revealed that 47% of AGS cases exhibited gastrointestinal symptoms [39], consistent with our results. We further detected that the odds of developing systemic reactions to meat after a tick bite increased by roughly 3% per year of age, with an average case age of 46 years. These results go in line with a systematic review from 2021 where the mean age of 229 AGS cases analyzed was 51 years [14]. Moreover, aging can potentially worsen allergic diseases, specially due to comorbidities and a decline in organ functionality [27].

Tick bites provide epidemiological evidence as a critical risk factor for AGS development, although they may not independently trigger the condition [40,41]. Our study revealed that age plays a role in both the development of immediate skin reaction to tick bites and the development of AGS-symptoms following meat consumption. Nevertheless, heightened effects associated with multiple tick bites in centre-north region on skin reactions were not reflected in the onset of AGS. This highlights the importance of interindividual variability in AGS, emphasizing the role of intrinsic risk factors such as genetic predisposition (family history of food allergy), blood type and immunological factors such as sIgE response linked to a history of previous allergies [42].

This study has several limitations for the interpretation of results. First, the number of participants is relatively small, and not all responded fully to the questionnaire, thus reducing statistical significance for model analysis and causing tendential rather than significant results. Second, potential information bias from self-reported data should be considered. Another point to acknowledge is the absence of data on tick species, pathogen infection, inquiries level of education and the time elapsed between tick removal and the development of skin lesions. Geographic granularity is restricted to autonomous communities or regions, which can comprise areas with heterogenous risk of tick exposure and differences in healthcare-seeking behaviors. Nevertheless, in Iberia, peak tick activity varies across species and some species are active year-round [43,44]. The administration of antibiotics in a prophylactic manner was based on the responses provided by survey participants, but without information on whether the antibiotics were self-administered by respondents in this context. Nevertheless, there are evidence supporting the use of single-dose doxycycline prophylaxis in endemic areas to reduce the risk of Lyme disease [45–47].

Overall, we conclude that the spread of ticks and TBDs are a rising social concern due to environmental, public health and economic factors. Therefore, raising awareness and increasing proactive measures in general population and healthcare providers should be a priority to mitigate the impact of TBDs. Medical professionals should be conscious of cumulative sensitization to tick bites and consider how geographical variations might affect the severity of reactions. The general reluctance to attend a health center post-tick exposure highlights the need for educational interventions on this matter. Moreover, limited knowledge among medical professionals about TBDs and AGS frequently leads to underdiagnosis, resulting in delayed treatment and worsened patient morbidity [16,40]. We should focus on older individuals as age appears to be an important risk factor not just for skin reactions post-tick exposure, but also for AGS. Overall, this study provides new insights that should be considered to improve TBDs surveillance and diagnostic strategies, as well as developing preventive measures to reduce tick bite exposure and AGS cases.

## Supplementary Material

Supplementary Fig1.jpg

Supplementary_Data_1.docx

Supplementary Tables.docx

Supplementary Fig2.png

Supplementary Data 2.xlsx

## Data Availability

The data reported in this paper are available within this article and the supplementary material. All data are also accessible upon reasonable request to the corresponding author.
