# Supplementary figures and images for: Public perspectives on tick bite exposure, healthcare visits and associated allergies in iberia
Source: Ann Med. 2025 May 3;57(1):2499028. doi: 10.1080/07853890.2025.2499028 (PMC12051554; doi:10.1080/07853890.2025.2499028)

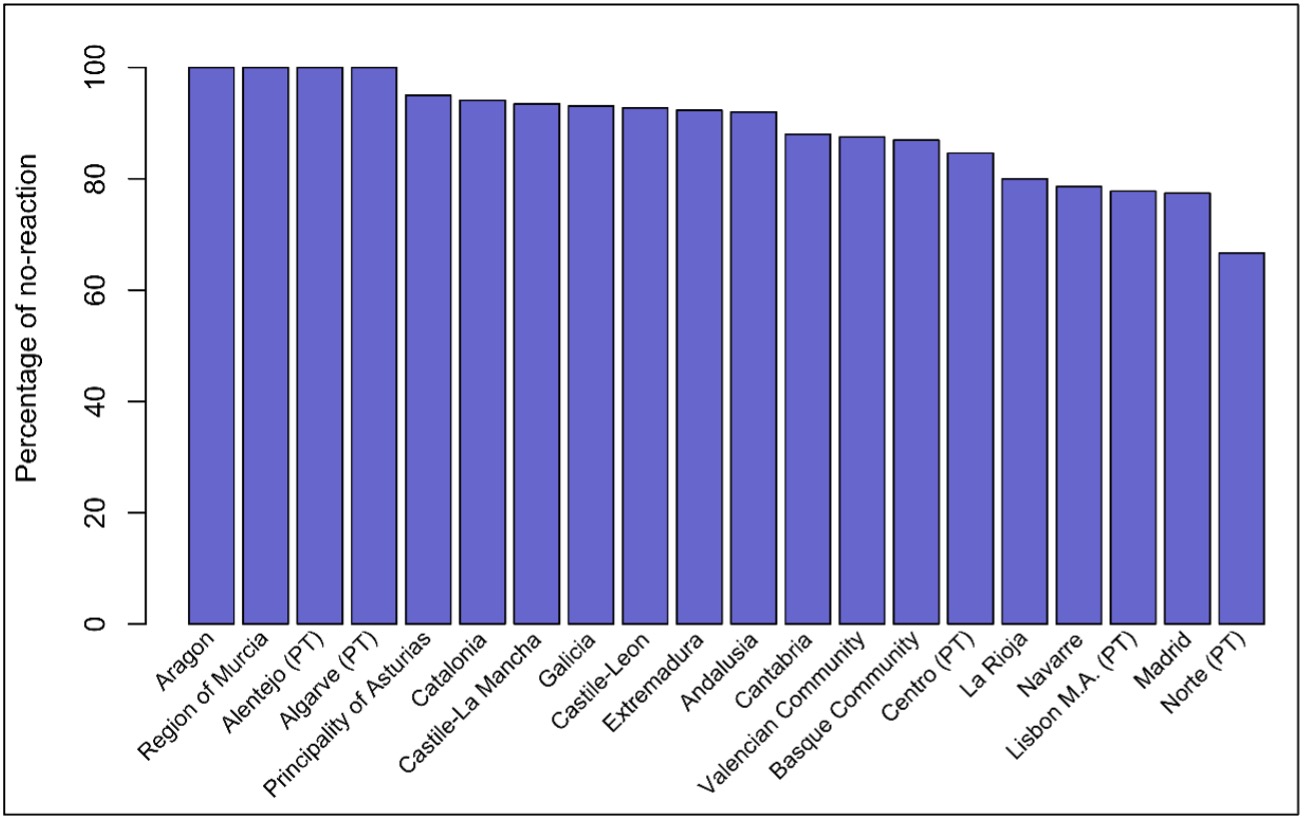

Supplement: Supplementary Fig1.jpg [file IANN_A_2499028_SM1142.jpg]

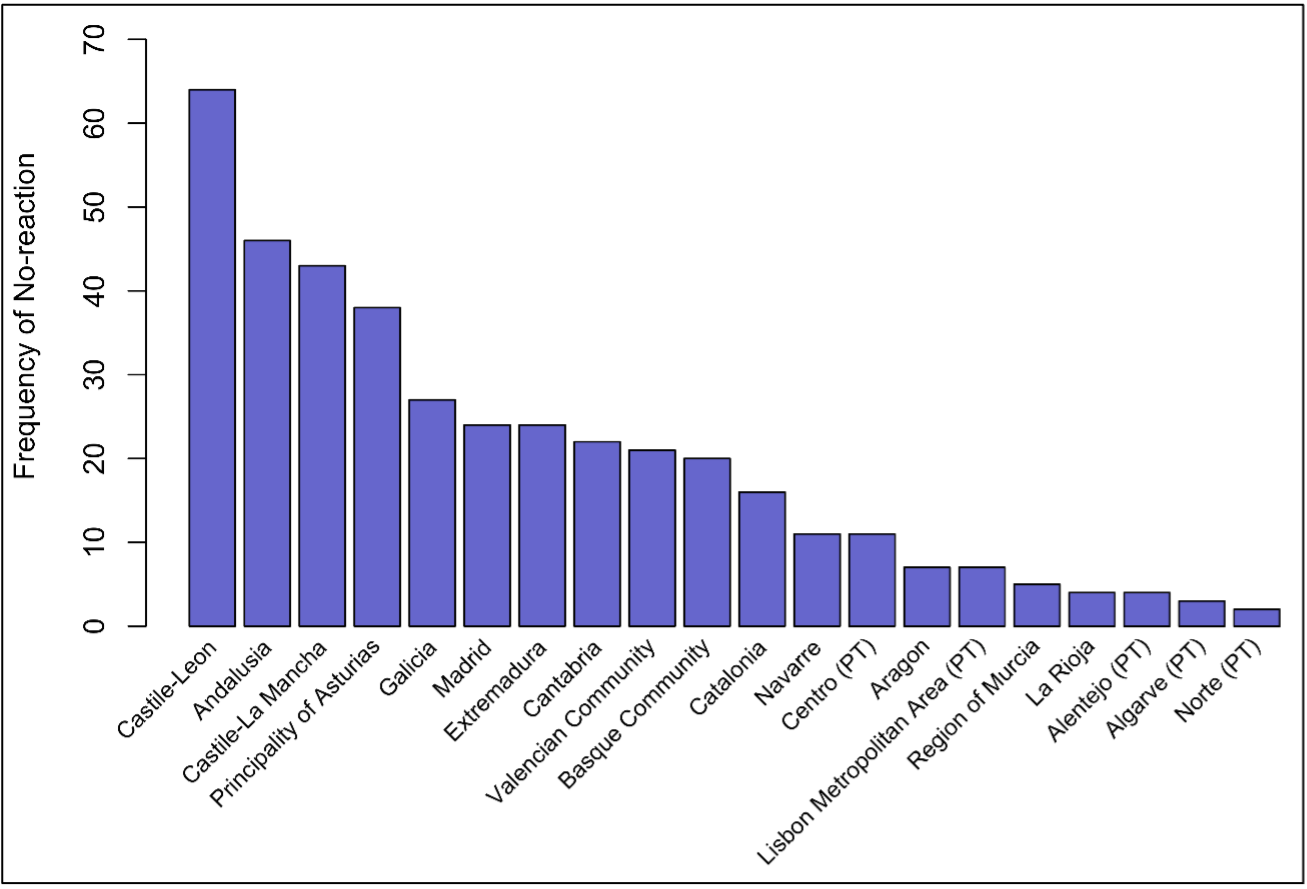

Supplement: Supplementary Fig2.png [file IANN_A_2499028_SM1139.png]
