# Supplementary material for: Public perspectives on tick bite exposure, healthcare visits and associated allergies in iberia
Source: Ann Med. 2025 May 3;57(1):2499028. doi: 10.1080/07853890.2025.2499028 (PMC12051554; doi:10.1080/07853890.2025.2499028)
Supplement: Supplementary_Data_1.docx [file IANN_A_2499028_SM1141.docx]

# Ticks and red meat allergy

Ticks are vectors of pathogens that affect the health of humans and animals. Its saliva contains multiple molecules that compromise the host's immune response, which can trigger inflammatory reactions. Among these molecules, we find the carbohydrate galactosa-α-1,3- galactosa (α-Gal), capable of producing antibodies in certain individuals, inducing allergies / anaphylactic reactions due to tick bites or consumption of red meat. This questionnaire was developed by the Institute of Game and Wildlife Research (IREC) within the scope of the BIOGAL project, which aims to collect information on the effects of tick bites and their possible association with red meat allergy (α-Gal Syndrome).

* Indicates a required question

## Sex *

*Check only one option.*


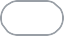
 Women
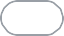
 Men

Other:

## Age *

1. Country of residence *

*Check only one option.*


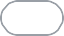
 Spain
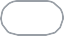
 Portugal

Other:

## Have you been bitten by a tick? *

*Check only one option.*


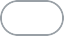
 Yes


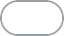
 No *Go to question 15*

1. Country where you **have been bitten** *

*Check only one option.*


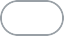
 Spain *Go to question 10*


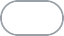
 Portugal *Go to question 7*


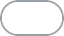
 Both *Go to question 8*


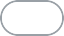
 None of the above *Go to question 11*

1. Other country where you **have been bitten**:

## Region of Portugal where you have been bitten:

*Check all that is applicable.*


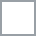
 Autonomous Region of Azores
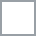
 Alentejo Region


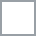
 Algarve


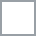
 Lisbon Metropolitan area
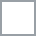
 Centro Region


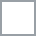
 Autonomous Region of Madeira
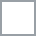
 Norte Region

*Go to question 11*

## Region of Portugal where you have been bitten:

*Check all that is applicable.*


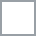
 Autonomous Region of Azores
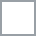
 Alentejo Region


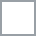
 Algarve


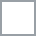
 Lisbon Metropolitan area
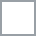
 Centro Region


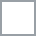
 Autonomous Region of Madeira
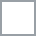
 Norte Region

## Autonomous Community where you have been bitten:

*Check all that is applicable.*


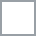
 Andalusia
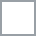
 Aragon


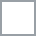
 Canary Islands
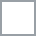
 Cantabria


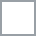
 Castile and León
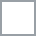
 Castilla-La Mancha
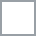
 Catalonia


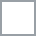
 Autonomous Region of Ceuta
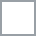
 Autonomous Region of Melilla
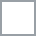
 Madrid


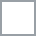
 Navarre
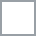
 Valencia


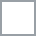
 Extremadura
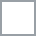
 Galicia


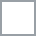
 Balearic Islands
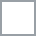
 La Rioja


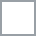
 Basque Country
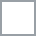
 Asturias


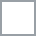
 Murcia

*Go to question 11*

## Autonomous Community where you have been bitten:

*Check all that is applicable.*


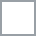
 Andalusia
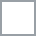
 Aragon


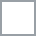
 Canary Islands
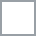
 Cantabria


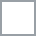
 Castile and León
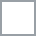
 Castilla-La Mancha
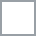
 Catalonia


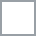
 Autonomous Region of Ceuta
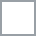
 Autonomous Region of Melilla
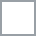
 Madrid


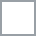
 Navarre
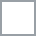
 Valencia


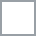
 Extremadura
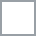
 Galicia


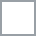
 Balearic Islands
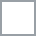
 La Rioja


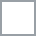
 Basque Country
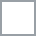
 Asturias


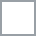
 Murcia

*Go to question 11*

1. Have you had allergies on the **bitten site**? *

*Check all that is applicable.*


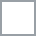
 No reaction
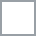
 Redness


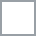
 Inflammation
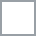
 Itch


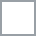
 Do not remember

## After being bitten by the tick, do you have any of the following reactions when you

**eat red meat** (1 to 6 hours after consumption)? *

*Check all that is applicable.*


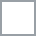
 No reaction


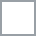
 Respiratory distress
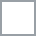
 Itch


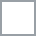
 Gastrointestinal symptoms
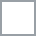
 Diarrhea


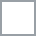
 Abdominal pain
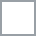
 Vomiting


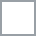
 Reflux


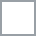
 Urinary incontinence
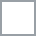
 Cardiovascular disease


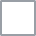
 Other:

## Have you been to the health center? *

*Check only one option.*


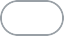
 Yes


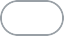
 No *Go to question 15*

1. At the health center were you diagnosed with?
2. If you have any extra information you want to share, take advantage of this space.
3. Do you allow this anonymous information to be used for research and scientific publications?

*Check only one option.*


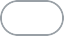
 Yes
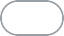
 No

Thank you very much for your collaboration.

Original questionnaire

# Las garrapatas y la alergia a la carne roja / As carraças e a alergia à carne vermelha

Las garrapatas son vectores de patógenos que afectan a la salud de humanos y animales. Su saliva contiene múltiples moléculas que comprometen la respuesta inmune del hospedador, pudiendo desencadenar reacciones inflamatorias. Entre estas moléculas, se encuentra el carbohidrato galactosa-α-1,3-galactosa (α-Gal), capaz de

producir anticuerpos en ciertos individuos, inductores de alergias/anafilaxias a través de picaduras de garrapatas o consumo de carne roja. Esta encuesta ha sido desarrollada

por el Instituto de Investigación en Recursos Cinegéticos (IREC) gracias al proyecto BIOGAL, el cual tiene como objetivo recopilar información sobre los efectos de las

picaduras de garrapatas y su posible asociación con la alergia a la carne roja (Síndrome de α-Gal).

As carraças são vectores de agentes patogénicos que afetam a saúde de humanos e animais. A sua saliva contém multiplas moléculas que comprometem a resposta imunitária do hospedeiro, podendo desencadear reações inflmatórias. Entre estas moléculas, encontramos o carbohidrato galactosa-α-1,3-galactosa (α-Gal), capaz de produzir anticorpos em certos individuos, indutores de alergias / reações anafiláticas pela picada de carraças ou consumo de carne vermelha. Este questionário foi

desenvolvido pelo Instituto de Investigação em Recursos Cinegéticos (IREC) no âmbito do projeto BIOGAL, que visa recolher informação sobre os efeitos das picadas de

carraças e a sua possível associação com a alergia à carne vermelha (Síndrome α-Gal).

* Indica uma pergunta obrigatória

## Sexo *

### Marcar apenas uma oval.

Mujer / Mulher

Hombre / Homem

Outra:

## Edad / Idade *

1. País de residencia / País de residência *

### Marcar apenas uma oval.

España / Espanha Portugal

Outra:

## Le ha picado alguna vez una garrapata? / Alguma vez foi picado por carraças? *

### Marcar apenas uma oval.


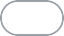
 Sí / Sim


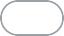
 No / Não *Avançar para a pergunta 15*

1. País donde ha **sido picado** / País onde **foi picado** *

### Marcar apenas uma oval.


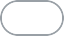
 España / Espanha *Avançar para a pergunta 10*


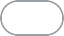
 Portugal *Avançar para a pergunta 7*


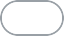
 Los dos anteriores / Os dois anteriores *Avançar para a pergunta 8*


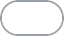
 Ninguno de los dos / Nenhum dos dois *Avançar para a pergunta 11*

1. Otro país donde ha **sido picado**: Outro país onde **foi picado**:

## Región de Portugal donde ha sido picado: Região de Portugal onde foi picado:

*Marcar tudo o que for aplicável.*


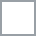
 Açores
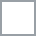
 Alentejo
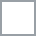
 Algarve


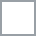
 Área Metropolitana de Lisboa
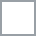
 Centro


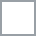
 Madeira
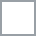
 Norte

*Avançar para a pergunta 11*

## Región de Portugal donde ha sido picado: Região de Portugal onde foi picado:

*Marcar tudo o que for aplicável.*


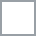
 Açores
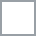
 Alentejo
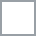
 Algarve


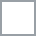
 Área Metropolitana de Lisboa
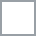
 Centro

Madeira Norte

## Comunidad autónoma donde ha sido picado: Comunidade Autónoma onde foi picado:

*Marcar tudo o que for aplicável.*

Andalucía Aragón Canarias Cantabria

Castilla y León

Castilla-La Mancha Cataluña/Catalunya

Ciudad Autónoma de Ceuta Ciudad Autónoma de Melilla Comunidad de Madrid

Comunidad Foral de Navarra Comunitat Valenciana

Extremadura Galicia

Islas Baleares La Rioja

País Vasco/Euskadi Principado de Asturías Región de Murcia

*Avançar para a pergunta 11*

## Comunidad autónoma donde ha sido picado: Comunidade Autónoma onde foi picado:

*Marcar tudo o que for aplicável.*

Andalucía Aragón Canarias Cantabria

Castilla y León

Castilla-La Mancha Cataluña/Catalunya

Ciudad Autónoma de Ceuta Ciudad Autónoma de Melilla Comunidad de Madrid

Comunidad Foral de Navarra Comunitat Valenciana

Extremadura Galicia

Islas Baleares La Rioja

País Vasco/Euskadi Principado de Asturías Región de Murcia

*Avançar para a pergunta 11*

1. Ha tenido alergias en la **zona de la picadura**? *

Teve alguma reação alérgica na **zona de picadura**?

*Marcar tudo o que for aplicável.*

Ninguna reacción / Nenhuma reação Enrojecimento / Vermelhidão

Inflamación / Inflamação Picor / Comichão

No me acuerdo / Não me lembro

1. Después de haber sido picado por la garrapata, cuando **consume carne roja** *

## tiene alguna de las siguientes reacciones (1 a 6h tras el consumo)?

Depois de ter sido picado pela carraça, quando **come carne vermelha** tem alguma das seguintes reações (1 a 6h após o consumo)?

*Marcar tudo o que for aplicável.*

Ninguna reacción / Nenhuma reação Estrés respiratorio / Stress respiratório Picor / Comichão

Síntomas gastrointestinales / Sintomas gastrointestinais Diarrea / Diarreia

Dolor abdominal / Dor abdominal Vómito

Reflujo / Refluxo

Retención o incontinencia urinaria / Retenção ou incontinência urinária Enfermedad cardiovascular / Doenças cardiovasculares

Outra:

## Ha asistido al centro de salud? / Foi ao centro de saúde? *

### Marcar apenas uma oval.

Sí / Sim

No / Não *Avançar para a pergunta 15*

1. En el centro de salud fue diagnosticado con? / No centro de saúde foi diagnosticado com?
2. Si tiene alguna información extra que quiera compartir, aproveche este espacio. Se tem alguma informação extra que queira partilhar, aproveite este espaço.
3. Autoriza a que esta información anónima sea utilizada para investigación y *

publicaciones científicas?

Autoriza a que esta informação anónima seja utilizada para investigação e publicações cientificas?

### Marcar apenas uma oval.

Sí / Sim No / Não

Muchas gracias por su colaboración. Muito obrigado pela sua colaboração.

Este conteúdo não foi criado nem aprovado pela Google.

[Formulários](https://www.google.com/forms/about/?utm_source=product&utm_medium=forms_logo&utm_campaign=forms)
