# Supplementary material for: Public perspectives on tick bite exposure, healthcare visits and associated allergies in iberia
Source: Ann Med. 2025 May 3;57(1):2499028. doi: 10.1080/07853890.2025.2499028 (PMC12051554; doi:10.1080/07853890.2025.2499028)
Supplement: Supplementary Tables.docx [file IANN_A_2499028_SM1140.docx]

**Supplementary Table 1.** Gender distribution of sampled respondents based on country of tick bite.

| **Gender** | **Portugal** | **Spain** | **Both** | **Total** |
| --- | --- | --- | --- | --- |
| Women | 17  (73.9%) | 151  (42.9%) | 4  (80%) | 172  (45.3%) |
| Men | 6  (26.1%) | 201  (57.1%) | 1  (20%) | 208  (54.7%) |

**Supplementary Table 2.** Age of the sampled inquiries according to country of tick bite.

| **Age group** | **Portugal** | **Spain** | **Both** | **Total** |
| --- | --- | --- | --- | --- |
| 18 - 24 | 1 | 24 | 0 | 25  (6.6%) |
| 25 - 34 | 8 | 85 | 5 | 98  (25.8%) |
| 35 - 44 | 5 | 62 | 0 | 67  (17.6%) |
| 45 - 54 | 3 | 100 | 0 | 103  (27.1%) |
| 55 - 64 | 2 | 58 | 0 | 60  (15.8%) |
| 65 or over | 4 | 23 | 0 | 27  (7.1%) |
| All | 23 | 352 | 5 | 380 |

**Supplementary Table 3.** Separation of administrative divisions in centre-north or centre-south regions to be included as a fixed factor in the statistical models.

| **Centre-north region** | **Centre-south region** |
| --- | --- |
| Galicia | Madrid |
| Principality of Asturias | Castile-La Mancha |
| Cantabria | Extremadura |
| Basque Community | Valencian Community |
| Navarre | Andalusia |
| La Rioja | Region of Murcia |
| Aragon | Algarve (PT) |
| Castile-Leon | Centro (PT) |
| Catalonia | Metropolitan Area of Lisbon (PT) |
| Norte (PT) | Alentejo (PT) |

Administrative division is represented by autonomous community in mainland Spain (NUTS2 2021) and statistical region in mainland Portugal (NUTS2 2013). The allocation of administrative divisions to regions was done to minimize the number of categories in the predictive variable for the statistical model.

**Supplementary Table 4.** Numerical output of the generalized linear model (GLM) for local skin reactions to tick bites.

|  | **Estimate** | **Std. error** | **z value** | ***p*-value** | **Odds ratio** |
| --- | --- | --- | --- | --- | --- |
| Intercept | -0.319 | 0.461 | -0.694 | 0.488 | 1.38 |
| Age | 0.024 | 0.010 | 2.426 | **0.015** | 1.02 |
| Gender (men) | -0.219 | 0.257 | -0.852 | 0.394 | 1.24 |
| Number of tick bites (multiple) | -0.686 | 0.382 | -1.795 | **0.073** | 1.99 |
| Region of tick bite (centre-south) | -0.481 | 0.255 | -1.892 | **0.059** | 1.62 |

This data comprises the estimated regression parameters, standard errors, z-values, *p*-values and odds ratios of the model. Fixed factors included age, gender (men/women), number of bites (single/multiple) and region of bite (centre-north/centre-south). The reference groups for each parameter are presented in parentheses.

**Supplementary Table 5.** Numerical output of the generalized linear model (GLM) for visiting health centres after a tick bite.

|  | **Estimate** | **Std. error** | **z value** | ***p*-value** | **Odds ratio** |
| --- | --- | --- | --- | --- | --- |
| Intercept | -3.321 | 0.651 | -5.101 | 3.38E-7 | 27.66 |
| Age | 0.004 | 0.011 | 0.385 | 0.700 | 1.00 |
| Gender (men) | 0.786 | 0.301 | 2.615 | **0.009** | 2.18 |
| Local skin reactions (single) | 0.615 | 0.364 | 1.69 | 0.091 | 1.09 |
| Local skin reactions (multiple) | 0.936 | 0.362 | 2.588 | **0.009** | 2.55 |
| Number of tick bites (single) | 1.083 | 0.407 | 2.661 | **0.007** | 2.95 |

This data comprises the estimated regression parameters, standard errors, z-values, *p*-values and odds ratios of the model. Fixed factors included age, gender (men/women), local skin reactions (none, single or multiple) and number of bites (single/multiple). The reference groups for each parameter are presented in parentheses.

**Supplementary Table 6.** Numerical output of the generalized linear model (GLM) for systemic symptomatology after mammalian meat consumption post-tick bites (α-Gal syndrome [AGS]-compatible signs).

|  | **Estimate** | **Std. error** | **z value** | ***p*-value** | **Odds ratio** |
| --- | --- | --- | --- | --- | --- |
| Intercept | -4.080 | 1.012 | -4.033 | 5.51E-5 | 59.15 |
| Age | 0.028 | 0.016 | 1.826 | **0.068** | 1.03 |
| Gender (men) | -0.207 | 0.414 | -0.500 | 0.617 | 1.23 |
| Number of tick bites (single) | 0.923 | 0.762 | 1.211 | 0.226 | 2.51 |
| Region of tick bite (centre-south) | -0.285 | 0.406 | -0.702 | 0.483 | 1.33 |

This data comprises the estimated regression parameters, standard errors, z-values, *p*-values and odds ratios of the model. Fixed factors included age, gender (men/women), number of bites (single/multiple) and region of bite (centre-north/centre-south). The reference groups for each parameter are presented in parentheses.
